# Examining D-dimer and Empiric Anti-coagulation in COVID-19-Related Thrombosis

**DOI:** 10.7759/cureus.26883

**Published:** 2022-07-15

**Authors:** Steven E Johnson, Eric Pai, Ashley Voroba, Nai-Wei Chen, Amit Bahl

**Affiliations:** 1 Emergency Medicine, Beaumont Health, Royal Oak, USA; 2 Emergency Medicine, Northwell Health, Manhasset, USA; 3 Biostatistics, Beaumont Health, Royal Oak, USA

**Keywords:** covid-19, lower extremity ultrasound, point of care ultrasound, bleeding events, microvascular complications, empiric anticoagulation, pulmonary embolism, deep vein thrombosis, venous thromboembolism, sars-cov-2

## Abstract

Objective

Thrombosis is thought to occur frequently in the setting of severe acute respiratory syndrome coronavirus 2 (SARS-CoV-2) infection. We aimed to elucidate the relationship between macro/microvascular thrombosis, D-dimer levels, and empiric anticoagulation in coronavirus disease 2019 (COVID-19).

Methods

This was an exploratory prospective, single-site, observational study. Adult emergency department patients with COVID-19 requiring hospitalization received a point-of-care lower extremity venous duplex ultrasound. The primary endpoint was thromboembolism and associated D-dimer level. Secondary endpoints included rates of micro and macro thrombotic complications as well as empiric anticoagulant use.

Results

Between January 13^th^ and April 12^th^ 2021, 52 patients were enrolled. Median D-dimer at presentation was 650 ng/mL (range 250-10,000 ng/mL) among patients with negative duplex studies. During hospitalization, 18 patients underwent 20 additional studies assessing for venous thromboembolism (VTE). These studies yielded one deep vein thrombosis (DVT) diagnosis. Among patients with negative studies median D-dimer was 1,246 ng/mL (range 329-10,000 ng/mL). Two patients experienced microvascular complications. Seven patients were started on empiric full dose anticoagulation.

Conclusion

While VTE remains a major concern amongst patients with COVID-19, the normal D-dimer cut off of >500 ng/mL likely should not be used to initiate further VTE workup. Additionally, moderately elevated D-dimer did not correlate strongly with microvascular complications and may not be relevant in the decision to initiate empiric anticoagulation.

## Introduction

The coronavirus disease 2019 (COVID-19) pandemic is an unprecedented public health event and many clinicians have struggled with how best to treat these complicated patients in the absence of robust scientific evidence. One complexity was that early in the pandemic we observed high rates of micro- and macrovascular thrombotic complications among COVID-19 patients, leading many physicians to question whether classic venous thromboembolism (VTE) management was sufficient [1-4]. More recent data suggests that the overall rate of VTE in COVID-19 may be similar to that of hospitalized patients pre-pandemic [5-10]. However, fearing thrombosis-related morbidity and mortality, many physicians have moved away from conventional guidelines regarding VTE workup and management in COVID-19 patients.

Specifically, for emergency department (ED) physicians, D-dimer is a quick test to screen COVID-19 patients for thrombotic disease. However, significant elevations in D-dimer levels are known to occur in severe acute respiratory syndrome coronavirus 2 (SARS-CoV-2) infection and it remains unclear how well these values correlate with the presence of VTE [11-13]. Some studies suggest that higher D-dimer thresholds, such as two to four times the normal cut-off value, could be used to trigger workup for VTE or initiation of empiric full anticoagulation (AC) among COVID-19 patients [14-16]. However, other studies suggest that even these higher cut-offs may have inadequate sensitivity in an ED population to rule out pulmonary embolism (PE) [17]. Several professional societies including the American College of Chest Physicians (CHEST), the National Institute of Health (NIH), and the American Society of Hematology have published guidelines regarding thrombosis in COVID-19. All of these societies recommend against the use of empiric full AC in the absence of confirmed or suspected VTEs [18-20]. Additionally, the NIH recommends against routine screening for VTE in COVID-19 patients regardless of the status of their coagulation markers [20]. Despite these recommendations, many physicians continue to use D-dimer levels to initiate VTE workup or start empiric full dose AC in COVID-19 patients. It is likely that these practice patterns have arisen due to an unclear message regarding the increased risk of VTE in COVID-19 and a lack of strong evidence of potential harm from these investigations and interventions.

In this pilot analysis, we aim to prospectively quantify the rate of VTE and microvascular complications amongst COVID-19 patients presenting to our ED and requiring admission. Our goal is to begin clarifying the role of a D-dimer level in informing ED physicians on the risk of thrombosis in this cohort.

## Materials and methods

Study design and setting

This was a prospective observational study assessing the relationship between D-dimer level and macro/microvascular complications. It was conducted at a single-site, tertiary care center with an annual ED census greater than 130,000 visits and 1,100 hospital beds. This study was approved by the Beaumont Health Institutional Review Board (#2020-303). Informed consent was obtained from all study subjects. The sample size was chosen based on the feasibility of the internal enrollment and possibility of performing exploratory analyses [21].

Selection of participants

The subject population consisted of a convenience sample of patients presenting to the ED. Study participation was voluntary and consent was obtained prior to enrollment. Patients eligible for the study were required to be at least 18 years of age and have laboratory-confirmed SARS-CoV-2 infection requiring hospitalization. Patients were excluded if they were on anticoagulation therapy prior to ED visit, pregnant, or incarcerated.

Study definitions

COVID-19 disease was defined as a positive result on reverse-transcriptase-polymerase-chain-reaction (RT-PCR) nasopharyngeal swab for SARS-CoV-2 in a patient that required hospitalization for acute illness. VTE was defined as lack of vein compressibility on lower extremity venous point of care ultrasound (POCUS) or comprehensive upper or lower extremity venous duplex or visualized thrombosis on chest computed tomography (CT). All POCUS exams were interpreted by ultrasound-fellowship trained physicians and other imaging was interpreted by board-certified radiologists. Microthrombotic complications were limited to myocardial infarction (MI) and ischemic stroke (CVA). MI was defined as any clinical documentation of suspected or confirmed MI, any EKG results consistent with ST elevation MI, or any rise in troponin (>0.3 ng/mL or an elevation >2x if elevated at baseline) without clinical documentation indicating a suspected secondary cause such as pulmonary embolism (PE) or demand ischemia. CVA was defined as any clinical documentation of transient ischemic attack or ischemic stroke, or imaging consistent with acute ischemic stroke. Empiric full AC was defined as physician documentation of empiric full dose AC, or if doses exceeded standard thromboprophylaxis ranges without documentation of a medical indication. Major bleeding events included any intracranial bleeding, clinically overt signs of hemorrhage resulting in hemodynamic compromise, gastrointestinal bleeding (GIB) resulting in hemodynamic instability or ≥2 units packed red blood cells (PRBC) transfusion in 24 hours. Minor bleeding events included clinically overt bleeding from intravenous (IV) sites, suspected GIB as documented by primary physician or confirmed on endoscopy not requiring ≥2 units PRBC transfusion in a 24-hour period, gross hematuria not associated with trauma, prolonged epistaxis >5 min or requiring intervention, all other clinically documented bleeding not leading to hemodynamic instability or ≥2 units PRBC transfusion in a 24-hour period.

Study procedure

Post-enrollment and within 24 hours of presentation to the ED, a standard focused two-point POCUS was performed and interpreted by a credentialed emergency physician competent in lower extremity venous imaging. The exam consisted of bilateral lower extremity compression ultrasound to determine presence or absence of thrombus with evaluation of the femoral vein and popliteal vein [22]. A combination of still images and clips were saved, and both reviewed and interpreted at the bedside as well as secondarily reviewed by the ultrasound director. The results of the POCUS were relayed to the treating physician in the ED.

All additional baseline and ongoing clinical data were collected from the electronic medical record (EMR). EMR data extraction was performed by EP and reviewed for accuracy by SJ. All individuals involved in EMR data collection were approved by the IRB and had undergone proper training regarding protected health information data collection. Relevant demographic data included: age, sex, and BMI. Additional baseline medical data were extracted including: admission vital signs, medical comorbidities, history of smoking, prior DVTs, current use of antiplatelet or anticoagulation, as well as relevant laboratory data. The EMR was queried for all relevant laboratory values, documentation of micro- or macrovascular complications, and any bleeding events. Additionally, medication administration was monitored to document the use of empiric full-dose anticoagulation. Monitoring of admitted patients’ charts continued until death or discharge.

Outcome measures

The primary outcome was the presence of VTE and associated D-dimer level. Secondary outcomes included the occurrence of macro/microvascular complications, frequency of empiric full dose anticoagulation use, and in-hospital bleeding events.

Statistical analysis

Continuous and categorical clinical characteristics were summarized as medians with interquartile ranges (IQRs) and frequencies (percentages), respectively. To establish D-dimer threshold levels in indicating the presence or absence of DVT, the diagnostic performance metrics of binary classification was assessed comparing to the comprehensive POCUS examination. Estimates of sensitivity, specificity, positive predictive value, negative predictive value, positive likelihood ratio, and negative likelihood ratio were reported with 95% confidence intervals (CIs). Subsequently, to further explore the association of numerical D-dimer levels and the DVT on POCUS examination, the logistic regression based on the profile penalized likelihood method was employed to estimate the effect of D-dimer levels along with the odds ratio and the corresponding 95% CI. All analyses were conducted with the use of R 4.0.2 (R Foundation for Statistical Computing) and SAS v9.4 (SAS Institute, Inc., Cary, NC, USA). 

## Results

Between January 13th 2021 and April 12th 2021, 54 patients were consented for the study. Two patients were excluded from the final analysis. The two exclusions resulted from one patient having their admission canceled after enrollment and being discharged from the ED and the other patient never had a D-dimer level drawn while in the emergency department.

Of 52 patients, the median age was 55 (IQR 43.5-64) with a slight majority of male patients (52%). 59.6% of patients had a BMI of less than 30. At presentation the median of the lowest systolic blood pressure (BP) was 116 (IQR 102.5-122.5) and the median pulse oximetry measurement was 93% (IQR 90-95). More than half of patients (30) presented with a saturation <94% on room air and of these, 10 had a saturation of ≤88% (Table 1). All patients were initially admitted to non-intensive care unit (ICU) beds from the ED. There was only one patient who was ever admitted to the ICU and a single death in this same patient.

**Table 1:** Clinical characteristics of patients with COVID-19 Abbreviations: BUN = blood urea nitrogen; FEU = fibrinogen-equivalent units §Data are reported as n (%) or median (interquartile range, IQR).

Variables^§^	All (n=52)	
Age, years	55.0	(43.5-64.0)
18 to 50-	21	(40.4)
50 to 65-	19	(36.5)
65 to 80-	9	(17.3)
≥ 80	3	(5.8)
Sex		
Male	27	(51.9)
Female	25	(48.1)
BMI, body mass index, kg/m^2^	28.2	(25.1-33.5)
< 30	31	(59.6)
≥ 30	21	(40.4)
SBP, systolic blood pressure, mmHg		
Lowest	116.0	(102.5-125.5)
Highest	144.0	(130.5-155.5)
DBP, diastolic blood pressure, mmHg		
Lowest	68.0	(56.5-73.0)
Highest	78.0	(66.5-88.0)
Pulse, beats per minute	104.0	(94.0-110.5)
Respiratory rate, breaths per minute	26.0	(22.5-32.0)
< 24	15	(28.9)
≥ 24	37	(71.1)
Blood oxygen saturation, %	93.0	(90.0-95.0)
≤ 88	10	(19.2)
88+ to 94-	20	(38.5)
≥ 94	22	(42.3)
Initial WBC count×10^9^/L	6.6	(4.7-9.5)
< 4	8	(15.4)
4 to 10	32	(61.5)
> 10	12	(23.1)
Initial Hemoglobin, g/dL	13.4	(12.4-14.5)
≤ 11	4	(7.7)
> 11	48	(92.3)
Initial Platelet count×10^9^/L	206.0	(168.5-257.5)
< 100	1	(1.9)
≥ 100	51	(98.1)
Initial Bun, mg/dL	16.0	(10.5-20.0)
Initial D-dimer, ng/mL FEU	665.0	(506.5-1154.0)
≤ 500	11	(21.1)
500 to 1000	24	(46.2)
> 1000	17	(32.7)
Highest D-dimer, ng/mL FEU	776.0	(537.0-1581.5)
≤ 500	9	(17.3)
500 to 1000	22	(42.3)
> 1000	21	(40.4)
Initial Creatinine, mg/dL	0.96	(0.84-1.28)
≤ 1.33	41	(78.8)
> 1.33	11	(21.2)
Highest Creatinine, mg/dL	0.96	(0.85-1.34)
≤ 1.33	39	(75.0)
> 1.33	13	(25.0)
Initial Troponin, ng/mL (n=49)	0.01	(0.01-0.02)
≤ 0.3	48	(98.0)
> 0.3	1	(2.0)
Highest Troponin, ng/mL (n=49)	0.01	(0.01-0.03)
≤ 0.3	48	(98.0)
> 0.3	1	(2.0)

The initial D-dimer among ED patients ranged from 250 to 10,000 ng/dL with a median of 665 (IQR 506.5-1154) at presentation. Forty percent of patients had a D-dimer >1,000 ng/mL at presentation (Table 1). Only one patient had a confirmed thrombotic event while in the ED. This patient had both a positive POCUS DVT study, identifying an asymptomatic popliteal vein thrombus, as well as a PE diagnosed by CT angiography (CTA). The D-dimer of this patient at presentation was 5,082 ng/mL. This single positive study among the 52 patients who underwent bilateral lower extremity duplex yielding a rate of 1.9% VTE among our ED cohort. A traditional D-dimer cut-off of 500 ng/mL was shown to have 100% (95% CI 3%-100%) sensitivity, but only 22% (95% CI 11%-35%) specificity to diagnose VTE in our population. D-dimer cutoffs of >500, >1,500 and >2,500 ng/mL showed positive likelihood ratios of 1.27 (95% CI 1.10-1.47), 7.29 (95% CI 3.66-14.50), and 17.00 (95% CI 5.67-50.96) respectively (Table 2). Additionally, amongst our ED patients, for every 500 unit increase in D-dimer the odds of DVT increased by 22% (OR [odds ratio] 1.22, 95%CI 0.98-1.54) (Table 3).

**Table 2:** Association of D-dimer and POCUS-DVT in ED Abbreviations: DVT = deep vein thrombosis; ED = emergency department; FEU = fibrinogen-equivalent units; POCUS = point of care ultrasound; DVT = deep vein thrombosis §Numerical D-dimer values were used in logistic regression. The profile penalized likelihood confidence intervals were shown.

D-dimer, ng/mL FEU		Odds Ratio^§^	(95% Confidence Interval)
For every 100-unit increase		1.04	(1.00-1.09)
For every 200-unit increase		1.08	(0.99-1.19)
For every 500-unit increase		1.22	(0.98-1.54)

**Table 3:** Diagnostic performance of each D-dimer threshold on POCUS-DVT in ED Abbreviations: DVT = deep vein thrombosis; ED = emergency department; PPV = positive predictive value; NPV = negative predictive value; LR+ = positive likelihood ratio; LR- = negative likelihood ratio; CI = confidence interval; NA = not available; FEU = fibrinogen-equivalent units

D-dimer thresholds	Sensitivity [95% CI]	Specificity [95% CI]	PPV [95% CI]	NPV [95% CI]	LR+ [95% CI]	LR- [95% CI]
(1) > 500 ng/mL FEU	1.00 (1/1) [0.03-1.00]	0.22 (11/51) [0.11-0.35]	0.02 [0.02-0.03]	1.00 [NA]	1.27 [1.10 to 1.47]	0 [NA]
(2) > 1500 ng/mL FEU	1.00 (1/1) [0.03-1.00]	0.86 (44/51) [0.74-0.94]	0.13 [0.07-0.22]	1.00 [NA]	7.29 [3.66-14.50]	0 [NA]
(3) > 2500 ng/mL FEU	1.00 (1/1) [0.03-1.00]	0.94 (48/51) [0.84-0.99]	0.25 [0.10-0.50]	1.00 [NA]	17.00 [5.67-50.96]	0 [NA]
(4) > 3000 ng/mL FEU	1.00 (1/1) [0.03-1.00]	0.94 (48/51) [0.84-0.99]	0.25 [0.10-0.50]	1.00 [NA]	17.00 [5.67-50.96]	0 [NA]

After admission, 18 of 52 (35%) patients underwent additional workup for VTE. Sixteen patients underwent CTA evaluating for PE; two of these patients, as well as two others, had an additional lower extremity ultrasound done during admission. Median D-dimer value among patients who underwent inpatient VTE workup was 1,246 ng/mL vs 626 ng/mL in the group who did not. Twenty-five (48%) of the admitted patients had a D-dimer value >500 ng/mL and did not undergo testing for VTE. Of 18 patients who did undergo testing, one additional DVT was diagnosed. This patient had a D-dimer of 897 at ED presentation that rose to 5,154 ng/mL at the time of DVT diagnosis. Of the 18 patients who underwent inpatient radiology study, we observed a VTE rate of 5.5% in our population. Amongst the entire study cohort, regardless of confirmed negative study, the overall rate of VTE was 3.8%.

Including ED POCUS studies, 70 exams evaluating for VTE were negative. At the time of the exams, we observed a range of D-dimer values from 250 to 10,000 ng/mL with an average of 1,279 ng/mL, a median of 696 ng/mL, and only 12 of these values below the cut-off of 500 ng/mL among negative studies.

Both patients who were diagnosed with VTEs in our cohort had D-dimer levels >5,000 ng/mL, or 10-fold the laboratory cut-off for normal, at the time of diagnosis. Among patients with negative inpatient imaging studies, the median D-dimer was 1,146 ng/mL with a range of 329 up to 10,000.

Only two patients met the criteria for microvascular complications in the form of one MI and one CVA with D-dimers of 2,313 and 5,154 ng/mL respectively. During admission, six patients were started on empiric full dose AC. The median D-dimer value was 2,313 ng/mL and 696 ng/mL among patients started on empiric full-dose AC and those not started on empiric full-dose AC, respectively. The single inpatient DVT that was diagnosed was in the empiric anticoagulation group after empiric AC had already been initiated. Additionally, one patient in this group experienced a minor bleeding event, specifically clinically overt bleeding from an IV site.

## Discussion

Unlike previous reports we observed a fairly low rate of VTE, 3.8%, among COVID-19 patients requiring admission. Among all 52 patients who underwent bilateral lower extremity doppler at ED presentation, the rate of VTE was even lower at 1.9%. Other recent publications have also shown that rates of VTE may not be as high as previously reported [5]. During the early phase of the pandemic, small studies reported high rates of thrombosis among COVID-19 patients admitted to ICUs causing alarm amongst the medical community and leading to guidelines to help prevent these complications from developing [3,23,24]. However in a recent meta-analysis, the overall rate of VTE among COVID-19 patients was 14.1% across eight studies, 7.9% amongst inpatients and 22.7% amongst patients admitted to the ICU [5]. Comparing this to pre-pandemic studies rates of VTE, among hospitalized non-ICU patients the rate of VTE is estimated to be between 2.8 to 5.6% and rates among ICU patients can be as high as 37% [6-10]. Another recent analysis looking at 445 COVID-19 patients presenting to the ED in whom the treating physician suspected PE observed a rate of 5.8% in this population [25]. Comparatively, previous studies have shown that the rate of PE in acute chronic obstructive pulmonary disease (COPD) exacerbation is 5.9% [26]. Overall, our findings as well as recent and historical data suggest that the rates of VTE in COVID-19 are likely similar to those observed among other non-COVID-19 patients with acute illness requiring hospitalization. As we observed no VTEs in COVID-19 patients with a D-dimer of less than 5,000 ng/mL, using the conventional cut-off of 500 ng/mL to initiate workup of VTE amongst COVID-19 patients is likely inappropriate. Among the 18 patients who underwent inpatient radiology studies for VTE, 10 (56%) of them had D-dimers of less than 1,500 at the time of the imaging. While it is unclear if there was a clinical indication for ordering these tests, given that the average D-dimer among patients who underwent inpatient imaging tests was much higher than those who did not, it is likely that this value influences the decisions of clinicians to initiate a VTE workup.

Given that the data suggests COVID-19 patients are similar to other patients with acute respiratory viral illnesses, when it comes to VTE, D-dimer results should not influence further VTE workup in cases with low clinical suspicion. Even though professional societies such as the NIH do not recommend using any coagulation markers to guide decision-making for VTE workup in COVID-19, it remains common practice as reflected within our cohort [12,18,20]. Given the incredibly low specificity reported in our study (22%) as well as previous literature, it seems clear that elevations above standard D-dimer cut-offs should not be used to trigger workup for VTE in COVID-19 without other clinical indications. In fact, we only observed a meaningful positive likelihood ratio (7.29) for the diagnosis of VTE once the D-dimer was >1,500 ng/mL.

While the exact D-dimer cut-off that is most appropriate amongst COVID-19 patients to initiate confirmatory testing for VTE is unclear, it is evident that using traditional D-dimer thresholds among patients with extremely low, or no clinical suspicion for PE, is likely inappropriate.

It is likely that early in the pandemic, the elevated concern for VTE among COVID-19 patients, as well as operational obstacles such as scarcity of personal protective equipment and sanitizing supplies led physicians away from conventional guidelines regarding VTE workup and management. There was a quick adoption of empiric anticoagulation strategies without confirmatory imaging despite recommendations against such strategies from professional societies [27-31]. Specifically, D-dimer shifted from being a screening test for patients with low to moderate clinical suspicion for PE to being utilized as a blanket screening tool for all comers to help guide physicians on the need to start empiric anticoagulation [14,16,17,32]. Presently, the practice of empiric anticoagulation persists despite a lack of substantiating evidence. For instance, we observed that the group of patients receiving empiric anticoagulation had a median D-dimer level approximately four-fold higher than the group who did not receive empiric AC, suggesting that D-dimer level may still be a factor weighed by physicians when deciding whether to initiate empiric AC in COVID-19. While concern for microvascular complications has classically been cited as the reason for initiation of empiric anticoagulation in COVID-19, our data, as well as recommendations from professional societies, suggest that D-dimer should not influence this decision [29,33]. Specific to our cohort, no microvascular complications were observed amongst inpatients with a D-dimer <2,300 ng/mL. Additionally, we only had one patient who required ICU admission and this patient did experience both a macrovascular VTE and a microvascular complication. The patient was intubated five days prior to diagnosis of a below-the-knee DVT. Microvascular complication was diagnosed on the basis of a rising troponin with a presumed myocardial infarction. However, these types of complications are not uncommon among ICU patients with other severe illness outside of COVID-19 [34,35]. As macro/microvascular complications were higher among ICU patients even before COVID-19, our data suggest that standard risk assessment is still the most prudent practice pattern even amongst COVID-19 patients. Additionally, while we only observed one non-severe bleeding event amongst the six patients in our AC cohort, it is prudent to establish a benefit of AC among COVID-19 patients prior to adopting an unproven and potentially harmful strategy.

Limitations

Our study had some limitations. Not all patients who experienced an increasing D-dimer level as an inpatient underwent additional VTE imaging. Therefore, it is possible that the rate of VTE is under-reported in our population. Additionally, this was a small sample size, single-site study and rates of VTE in COVID-19 may vary based on other factors such as race, sex, variant type, and time since symptom onset, none of which we were able to evaluate for here. Only one patient in our cohort was admitted to the intensive care unit and given the higher rates of VTE among ICU patients our findings may be less accurate in this population. This study only examined inpatients admitted for COVID-19 and the rates of VTE may be different among patients who are discharged from the ED. Finally, as treatments for COVID-19 continue to evolve, it is unclear how these will affect D-dimer levels and the rates of VTE among hospitalized COVID-19 patients.

## Conclusions

Although DVT and PE remain major concerns amongst patients diagnosed with COVID-19, emerging data, including findings from our cohort, suggest that the rates of VTE in acute COVID-19 may be similar to those observed in other conditions requiring hospitalization. Additionally, given the significant elevations of D-dimer values seen in acute SARS-CoV-2 infection, the normal D-dimer cut-off of >500 ng/mL likely should not be used as a trigger to initiate further workup for VTE, especially among ED patients. However, a significantly elevated D-dimer may be more helpful. Mildly to moderately elevated D-dimer values did not correlate strongly with microvascular complications and may not be relevant in the decision to initiate empiric full-dose anticoagulation outside of another indication. Despite the concern for VTE in COVID-19, it appears as though previously validated strategies for working up potential VTEs as well as initiation of anticoagulation are likely still the best recommendations. Further research is needed to determine if specific subsets of COVID-19 patients may benefit from D-dimer screening or empiric anticoagulation.
